# Timing of Major Postoperative Bleeding Among Patients Undergoing Surgery

**DOI:** 10.1001/jamanetworkopen.2024.4581

**Published:** 2024-04-02

**Authors:** Alex L. E. Halme, Pavel S. Roshanov, Sara V. Tornberg, Lauri I. Lavikainen, P. J. Devereaux, Kari A. O. Tikkinen

**Affiliations:** 1 Faculty of Medicine, University of Helsinki, Helsinki, Finland; 2 Population Health Research Institute, McMaster University, Hamilton, Ontario, Canada; 3 Department of Medicine, Western University, London, Ontario, Canada; 4 Department of Epidemiology and Biostatistics, Western University, London, Ontario, Canada; 5 Outcomes Research Consortium, Cleveland, Ohio; 6 Department of Urology, University of Helsinki and Helsinki University Hospital, Helsinki, Finland; 7 Department of Health Research Methods, Evidence and Impact, McMaster University, Hamilton, Ontario, Canada; 8 Department of Medicine, McMaster University, Hamilton, Ontario, Canada; 9 Department of Surgery, South Karelian Central Hospital, Lappeenranta, Finland

## Abstract

**Importance:**

Although major bleeding is among the most common and prognostically important perioperative complications, the relative timing of bleeding events is not well established. This information is critical for preventing bleeding complications and for informing the timing of pharmacologic thromboprophylaxis.

**Objective:**

To determine the timing of postoperative bleeding among patients undergoing surgery for up to 30 days after surgery.

**Design, Setting, and Participants:**

This is a secondary analysis of a prospective cohort study. Patients aged 45 years or older who underwent inpatient noncardiac surgery were recruited in 14 countries between 2007 and 2013, with follow-up until December 2014. Data analysis was performed from June to July 2023.

**Exposure:**

Noncardiac surgery requiring overnight hospital admission.

**Main Outcomes and Measures:**

The primary outcome (postoperative major bleeding) was a composite of the timing of the following bleeding outcomes: (1) bleeding leading to transfusion, (2) bleeding leading to a postoperative hemoglobin level less than 7 g/dL, (3) bleeding leading to death, and (4) bleeding associated with reintervention. Each of the components of the composite primary outcome (1-4) and bleeding independently associated with mortality after noncardiac surgery, which was defined as a composite of outcomes 1 to 3, were secondary outcomes.

**Results:**

Among 39 813 patients (median [IQR] age, 63.0 [54.8-72.5] years; 19 793 women [49.7%]), there were 5340 major bleeding events (primary outcome) in 4638 patients (11.6%) within the first 30 days after surgery. Of these events, 42.7% (95% CI, 40.9%-44.6%) occurred within 24 hours after surgery, 77.7% (95% CI, 75.8%-79.5%) by postoperative day 7, 88.3% (95% CI, 86.5%-90.2%) by postoperative day 14, and 94.6% (95% CI, 92.7%-96.5%) by postoperative day 21. Within 48 hours of surgery, 56.2% of major bleeding events, 56.2% of bleeding leading to transfusion, 56.1% of bleeding independently associated with mortality after noncardiac surgery, 51.8% of bleeding associated with hemoglobin less than 7 g/dL, and 51.8% of bleeding associated with reintervention had occurred.

**Conclusions and Relevance:**

In this cohort study, of the major postoperative bleeding events in the first 30 days, more than three-quarters occurred during the first postoperative week. These findings are useful for researchers for the planning future clinical research and for clinicians in prevention of bleeding-related surgical complications and in decision-making regarding starting of pharmacologic thromboprophylaxis after surgery.

## Key Points

**Question:**

What is the timing of major bleeding events occurring after surgery?

**Findings:**

In this cohort study of 39 813 patients, of all major bleeding events that occurred within the first 30 days after surgery, 42.7% occurred within 24 hours of surgery, 77.7% by postoperative day 7, 88.3% by postoperative day 14, and 94.6% by postoperative day 21.

**Meaning:**

These findings suggest that of all major bleeding events within 30 days after surgery, most occur within 1 week after surgery; these findings could aid clinicians in preventing bleeding-related surgical complications and in decision-making regarding the start of pharmacologic thromboprophylaxis after surgery.

## Teaser

This cohort study examines the timing of postoperative bleeding among patients undergoing surgery for the first 30 days after the procedure.

## Introduction

More than 300 million patients undergo surgery annually worldwide.^1^ Bleeding is among the most common surgical complications and is associated with blood transfusion, reintervention, organ injury, and death, as well as increased costs.^2,3,4^ Surgical patients often receive prophylactic or therapeutic anticoagulant and antiplatelet medications. Postoperative bleeding risk, which dissipates after surgery, is relevant for decisions about when to use these agents.

Understanding the risk and timing of postoperative bleeding is important for patient care for several reasons. For example, in addition to bleeding, patients undergoing surgery are at risk of thromboembolism and are, therefore, often prescribed thromboprophylaxis.^5,6,7,8,9,10^ The use of pharmacologic thromboprophylaxis involves a trade-off between a reduction in risk of venous thromboembolism (VTE) and increase in the risk of bleeding. Understanding of the timing of bleeding is critical for making decisions about the starting time and duration of pharmacologic prophylaxis, as well as to anticipate and prevent these complications.

To our knowledge, there are no systematic summaries on the timing of postoperative bleeding in surgery. We, therefore, undertook this secondary analysis of the large, international prospective VISION (Vascular Events in Noncardiac Surgery Patients Cohort Evaluation) study to determine the evolution of bleeding risk over time in the period immediately after major surgery.^11^ The results of this secondary analysis of the VISION study will also inform clinicians and practice guidelines when deciding on perioperative practices, including surgical thromboprophylaxis.

## Methods

### Study Design and Setting

This is a secondary analysis of the VISION study, a prospective cohort study of patients aged 45 years or older who had inpatient noncardiac surgery between 2007 and 2013 at 28 centers in 14 countries.^12^ The ethics review board at each participating center approved the VISION study protocol. The details and methods of the VISION study have been described previously.^11^ We followed the Strengthening the Reporting of Observational Studies in Epidemiology (STROBE) reporting guidelines for cohort studies.^13^

All eligible patients underwent noncardiac surgery requiring overnight hospital admission after surgery. Study personnel identified potential participants through daily screening of patient lists in preoperative assessment clinics, surgical lists from the same and previous day, lists on surgical wards and in intensive care units, and in preoperative holding areas. Enrolled patients answered a series of questions about their medical and social history. Patients provided written informed consent. Research personnel reviewed medical records for further background history, noted outcome events throughout the hospital stay, and conducted a follow-up telephone interview with the patient or their next of kin 30 days after surgery. Research staff obtained further documentation, including dates of events, if the interview indicated the occurrence of an outcome event. Investigators reviewed and approved data at each site. Research personnel submitted case report forms and supporting documentation directly to the coordinating center. Data monitoring involved central data consistency checks, statistical monitoring, and on-site monitoring for all centers.

### Outcomes

We aimed to describe the timing of bleeding within 30 days after surgery. We based our outcomes on diagnostic criteria for bleeding, which have been shown to be independently associated with mortality, including bleeding independently associated with mortality after noncardiac surgery (BIMS).^14,15^ Our primary outcome (ie, postoperative major bleeding) was a composite of the timing of the following bleeding outcomes: (1) bleeding leading to any transfusion of red blood cells, (2) bleeding leading to a postoperative hemoglobin level less than 7 g/dL (to convert hemoglobin to grams per liter, multiply by 10), (3) bleeding leading directly to death, and (4) bleeding associated with reintervention. Each of the components of the primary composite outcome (1-4) and BIMS,^14,15^ which was defined as a composite of outcomes 1 through 3, were secondary outcomes.

### Approach to Missing Data

In the primary analysis, we excluded patients with missing data regarding the timing of surgery or day of bleeding. If the time of surgery and the date of bleeding were known but the time was missing, we estimated the time of bleeding as noon (as this ensures that we are at most 12 hours off from the true time of bleeding) and did not exclude the patient. Our secondary analyses excluded patients with incomplete outcome or covariate data.

### Statistical Analysis

We wrote a statistical analysis plan before conducting the analyses. To determine the timing of bleeding, we calculated the absolute risk of each outcome, as well as the cumulative relative frequency (ie, the cumulative distribution function) of bleeding events on each day up to postoperative day 30. We calculated the absolute risk of an event occurring on each day up to postoperative day 30 by dividing the number of events on any given day with the number of patients alive on that day. We calculated the cumulative proportion of bleeding events by dividing the number of events that occurred between the time of operation and a specific time point with the total number of events that occurred during the total 30-day follow-up period. We also conducted sensitivity analyses by patient recruitment year and surgical specialty.

In an exploratory secondary analysis, we used logistic regression to examine the associations of early (vs late) postoperative major bleeding with patient characteristics, preoperative medications, study center, year of patient recruitment, and the type of surgery. Because there is no consensus on what counts as early or late postoperative bleeding, through discussion and consensus building, we defined early bleeding as postoperative major bleeding occurring in the first 48 hours after surgery and late bleeding as postoperative major bleeding occurring after the first 48 hours. Because the secondary analysis was a purely exploratory post hoc analysis with no causal interpretation, we did not adjust for multiple testing and used a significance level of *P* < .05 and 2-sided hypothesis tests. We conducted analyses in June and July of 2023 using R statistical software version 4.2.3 (R Project for Statistical Computing).^16^

## Results

**Figure 1.**
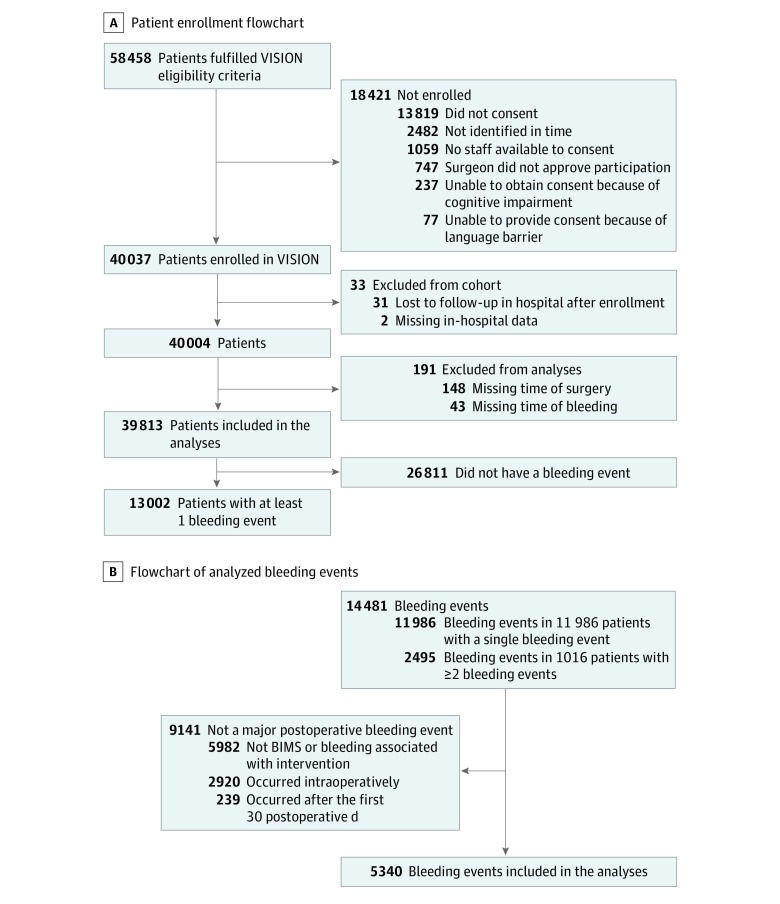
Study Flowcharts A, Flowchart shows enrollment of the analysis population in the Vascular Events in Noncardiac Surgery Patients Cohort Evaluation (VISION) study. B, Flowchart of analyzed bleeding events. Bleeding independently associated with mortality after noncardiac surgery (BIMS) is a composite outcome of postoperative transfusion, hemoglobin less than 7 g/dL (to convert hemoglobin to grams per liter, multiply by 10) and bleeding directly leading to death.

Of a total of 40 004 patients, 39 813 (99.5%) had sufficient data and were included in the analyses (Figure 1A). We excluded 191 patients (0.5% of patients enrolled and 1.5% of those with a bleeding event) from our analyses owing to missing data on the timing of surgery or timing of bleeding. Although 14 481 bleeding events (13 002 patients) were reported in the VISION study, we did not count 9141 (minor) bleeding events as major postoperative bleeding because they did not fulfill our definition. Finally, our analyses included 5340 major bleeding events from 4638 of 39 813 patients (11.6%) (Figure 1B).

The Table describes the cohort characteristics. The median (IQR) age was 63.0 (54.8-72.5) years, and 19 793 patients (49.7%) were women. The most common surgical categories were general surgery (8187 patients [19.7%]), major orthopedics (6959 patients [16.8%]), major urology or gynecology (4970 patients [12.0%]), and low-risk procedures (15 228 patients [36.7%]). Of the patients, 20 141 (50.6%) had hypertension, and 8383 (21.1%) had diabetes. Within 24 hours before surgery, 2816 patients (7.1%) had used acetylsalicylic acid, ticlopidine, or clopidogrel, and 5322 (13.4%) had used prophylactic subcutaneous antithrombotic agents.

**Table.** Patient Clinical and Surgical Characteristics

Characteristics	Patients, No. (%) (N = 39 813)
Age range, y	
45-64	22 022 (55.3)
65-74	10 116 (25.4)
≥75	7675 (19.3)
Sex	
Male	20 020 (50.3)
Female	19 793 (49.7)
Body mass index^a^	
<18.5	1436 (3.61)
18.5-24.9	13 390 (33.6)
25.0-29.9	13 010 (32.7)
30.0-39.9	8749 (22.0)
≥40	1418 (3.56)
Type of surgery	
Major general surgery	8187 (19.7)
Major orthopedic surgery	6959 (16.8)
Major urology or gynecology	4970 (12.0)
Major vascular surgery	2654 (6.4)
Major neurosurgery	2329 (5.6)
Major thoracic surgery	1191 (2.9)
Other (low-risk only) surgery	15 228 (36.7)
Preoperative medications	
NSAID and/or COX-2 inhibitor <24 h	3690 (10.0)
NSAID and/or COX-2 inhibitor 7 d to >24 h	4791 (12.5)
ASA, ticlopidine, or clopidogrel <24 h	2816 (7.1)
ASA, ticlopidine, or clopidogrel 7d to >24 h	6964 (17.5)
Prophylactic SC AA <24 h	5322 (13.4)
Prophylactic SC AA 7 d to >24 h	2935 (7.4)
Therapeutic AA or DOAC <24 h	544 (1.4)
Therapeutic AA or DOAC 7 d to >24 h	1595 (4.0)
Statin <24 h	8549 (21.5)
Statin 7 d to >24 h	10 727 (26.9)
Postoperative medications	
NSAID and/or COX-2 inhibitor 3 d^b^	14 195 (35.7)
NSAID and/or COX-2 inhibitor at discharge	7068 (17.8)
ASA, ticlopidine, or clopidogrel 3 d	6658 (16.7)
ASA, ticlopidine, or clopidogrel at discharge	7723 (19.4)
Prophylactic SC AA 3 d	18 833 (47.3)
Prophylactic SC AA at discharge	7419 (18.6)
Therapeutic AA or DOAC 3 d	1320 (3.32)
Therapeutic AA or DOAC at discharge	536 (1.35)
Statin 3 d	9603 (24.1)
Statin at discharge	10 573 (26.6)
Comorbidities	
History of hypertension	20 141 (50.6)
History of peripheral vascular disease	3193 (8.0)
History of cardiac arrest	360 (0.9)
History of diabetes	8383 (21.1)
History of deep vein thrombosis and/or pulmonary embolism	1436 (3.6)
Total major bleeding events^c^	5340

Abbreviations: AA, antithrombotic agent; ASA, acetylsalicylic acid; COX-2, cyclooxygenase-2; DOAC, direct oral anticoagulant; NSAID, nonsteroidal anti-inflammatory drug; SC, subcutaneous.

^a^ Body mass index is calculated as weight in kilograms divided by height in meters squared. Data were missing for 1810 patients (4.55%).

^b^ Refers to any use within first 3 postoperative days.

^c^ Only refers to postoperative bleeding events classified as bleeding independently associated with mortality after noncardiac surgery or bleeding associated with reintervention.

**Figure 2.**
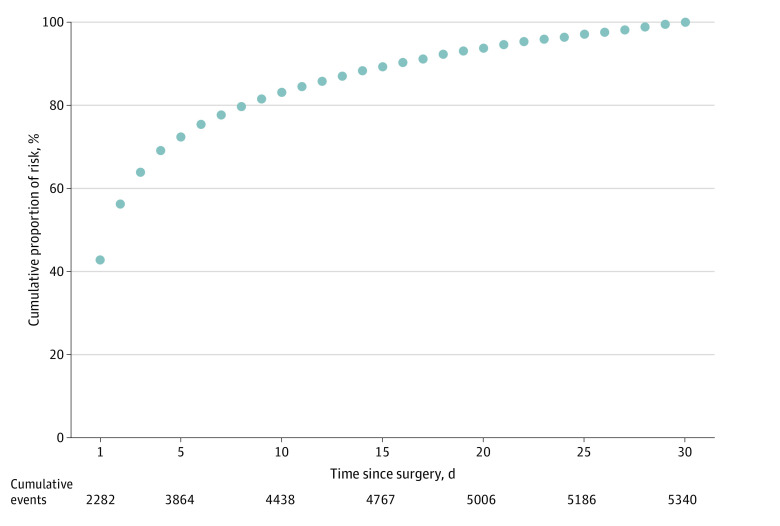
Cumulative Relative Frequency of Major Bleeding Events Within the First 30 Postoperative Days Major bleeding is a composite outcome of postoperative transfusion, hemoglobin less than 7 g/dL (to convert hemoglobin to grams per liter, multiply by 10), or bleeding leading directly to death.

**Figure 3.**
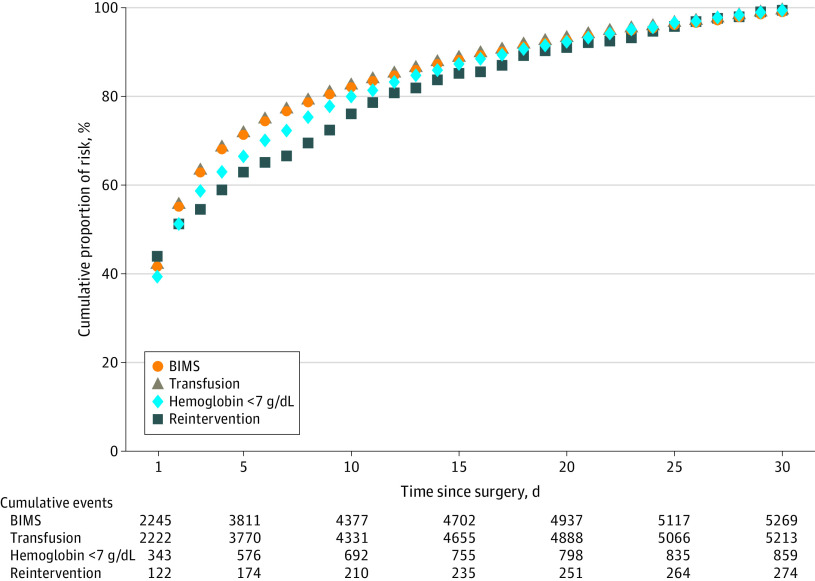
Cumulative Relative Frequency of Bleeding Independently Associated With Mortality After Noncardiac Surgery (BIMS), Transfusion of 1 or More Units of Red Blood Cells, Nadir Hemoglobin Less Than 7 g/dL and Bleeding Associated With Reintervention Within the First 30 Postoperative Days BIMS is a composite outcome of postoperative transfusion, hemoglobin less than 7 g/dL (to convert hemoglobin to grams per liter, multiply by 10), or bleeding leading directly to death.

Of the 5340 major bleeding events, 42.7% (95% CI, 40.9%-44.6%) occurred within 24 hours postoperatively, 77.7% (95% CI, 75.8%-79.5%) occurred by postoperative day 7, 88.3% (95% CI, 86.5%-90.2%) occurred by day 14, and 94.6% (95% CI, 92.7%-96.5%) occurred by day 21 (Figure 2). Figure 3 illustrates separately the cumulative proportion of BIMS, bleeding associated with reintervention, and postoperative hemoglobin less than 7 g/dL and transfusions by time. Within 48 hours of surgery, 56.2% of major bleeding events, 56.2% of bleeding leading to transfusion, 56.1% of bleeding independently associated with mortality after noncardiac surgery, 51.8% of bleeding associated with hemoglobin less than 7 g/dL, and 51.8% of bleeding associated with reintervention had occurred. The absolute risks (events) were 13.2% (5269 events) for BIMS, 13.1% (5213 events) for transfusions, 2.2% (859 events) for postoperative hemoglobin less than 7 g/dL, 0.69% (274 events) for reintervention, and 0.06% (25 events) for death (eFigure 1 in Supplement 1). Bleeding associated with reintervention and postoperative hemoglobin of less than 7 g/dL occurred later than transfusion (Figure 3). The cumulative proportions by time of the primary outcome (major bleeding), BIMS, and transfusion were similar (Figure 3 and eTable 1 in Supplement 1).

Additional details on the risks of major bleeding, including figures with absolute risk estimates, stratification by surgical specialty and year of surgery, and 95% CIs for primary and secondary outcomes, are shown in eTable 2 and eFigures 2, 3, and 4 in Supplement 1. Information regarding bleeding leading to death among 25 patients is shown in eTable 1 in Supplement 1. Results of the exploratory secondary analysis are shown in eTable 3 in Supplement 1.

## Discussion

This secondary analysis of the large, prospective VISION cohort study describes the postoperative timing of bleeding within 30 days after surgery. We found that 42.7% of major bleeding events occurred within the first 24 hours after surgery, 77.7% within 7 days, 88.3% within 14 days, and 94.6% within 21 days (Figure 2). We also found that bleeding associated with reintervention usually occurred later than BIMS.

### Relationship to Prior Work

Despite the major importance of postoperative bleeding, its timing has not been well established. In the existing literature, studies on the timing of bleeding were limited to a single procedure,^17,18,19^ based on data from randomized clinical trials with strict inclusion criteria,^20,21,22^ or had fewer events leading to lower precision in their estimates.^17,23,24,25^ All of these points limit the applicability of earlier evidence to surgical practice.

In a Japanese retrospective study,^17^ 489 patients undergoing submucosal endoscopic dissection had a bleeding event, defined as emergency endoscopy owing to clinical symptoms (hematemesis, melena, or decrease in hemoglobin levels >2 g/dL). The postoperative bleeding events peaked on the first postoperative day, but unlike in our study, those authors also found a secondary peak around day 7. Although in our study almost 80% of bleeding events occurred by postoperative day 7, the corresponding estimate in that study was approximately 60%. In a retrospective British study,^19^ the authors identified 14 bleeding events among 805 patients undergoing thyroid surgery. Of these events, 11 occurred during the first postoperative day. These differences between the 2 previous studies and our study may be explained by the special characteristics of the surgical procedures performed (both studies), different outcomes used for bleeding (both studies), and small sample size (British study).^17,19^

In 2 large randomized studies,^20,21^ among patients undergoing noncardiac surgery, the risk of bleeding diminished substantially after 8 to 10 days. In those studies, approximately 90% of major bleeding events occurred by day 7 compared with our estimate of 77.7%. Both of those trials recruited patients undergoing major surgical procedures with several risk factors for both bleeding and thrombosis. Thus, the results from those trials are likely less generalizable than our estimates for patients with a more typical risk profile across various surgical fields. Our study population was a more representative sample including patients with both major and low-risk surgery, who were included without requirements for risk factors for bleeding or cardiovascular events.

### Implications of the Findings

Despite the high volume of noncardiac surgery,^11^ major complications such as bleeding associated with reintervention or BIMS remain a major source of morbidity.^26^ Our work has established that, of all major bleeding events within the first 30 days after surgery, 42.7% occurred during the first postoperative day and 77.7% occurred during the first week after surgery. An understanding of the timing of bleeding is crucial in surgical practice to anticipate and prevent these complications.

The results of our study can be useful for researchers designing clinical trials. Because most bleeding events typically occur soon after surgery, an extended follow-up of several months for these complications is probably not warranted. In addition, researchers can use our results when summarizing the absolute risks of major bleeding between different studies with various follow-up times.

Although prior work exists on procedure-specific risks of thrombosis and bleeding in surgery (with variable certainty evidence),^27,28,29,30,31,32,33^ the optimal starting time and duration of thromboprophylaxis remains unclear owing to insufficient statistical power of existing randomized trials in the field and the changing nature of surgery (eg, earlier mobilization and less-invasive surgery).^34,35^ This has contributed to substantial practice variation in thromboprophylaxis worldwide.^36,37,38^ Because decisions regarding thromboprophylaxis in noncardiac surgery often represent a trade-off between decreased risk of VTE and increased risk of bleeding,^39^ an understanding of the postoperative timing of major bleeding plays a major role in assessing the risks and benefits associated with thromboprophylaxis. Our results, in conjunction with earlier results on the timing of VTE,^10^ suggest that the further the patient is from surgery, the greater the net benefit of thromboprophylaxis. Initiation of pharmacological thromboprophylaxis on the day of surgery may often not be needed, especially in patients without high risk of VTE.

An understanding of the timing of bleeding and baseline risks of bleeding in surgery are useful when determining when to resume discontinued medications after surgery. Clinicians may also use our results in conjunction with baseline risks of surgical procedures. For example, if there is a need to start postsurgical thromboprophylaxis with anticoagulation or a need to resume discontinued antithrombotic agent after surgery, our results give guidance regarding what proportion of the total 30-day bleeding risk has accumulated by a certain day that can guide trade-off of the risks and benefits of (re-)starting antithrombotic medications after surgery. Randomized trials of different thromboprophylaxis regimens in representative patient populations are needed to fully rationalize global thromboprophylaxis practice in noncardiac surgery.^40^

### Strengths and Limitations

This study has many strengths. First, this study is based on a prospective and representative international sample of more than 40 000 noncardiac surgical patients (with <0.1% lost to follow-up) undergoing procedures across various fields.^11^ Second, we included more than 5000 major bleeding events, leading to high precision in our estimates, and used patient-important and validated outcome measures.^14^ Third, we followed a statistical analysis plan that was drafted before analyses.

This study also has limitations. Patients enrolled in VISION underwent surgery between October 2008 and December 2013. Since the start of the VISION study, there has been a trend toward reduced transfusions^41,42^ and increased use of tranexamic acid, especially in orthopedic surgery.^43,44^ Because absolute risks of bleeding events are likely sensitive to changes in these practices, our study may overestimate the absolute risk estimates compared with current surgical practice. However, we conjectured that even if the incidence of postoperative bleeding complications were to decrease, this would likely not have an important impact on the timing of the events. Our sensitivity analysis for patient recruitment year (eFigure 3 in Supplement 1) supports this: the results are consistent across time. In addition, newer antithrombotic medications have also been introduced, especially in orthopedic surgery, but without major differences in bleeding risk compared with low-molecular-weight heparin.^45^ Furthermore, we were unable to control for use of postoperative thromboprophylaxis.

## Conclusions

In this cohort study, of the major postoperative bleeding events in the first 30 days, 42.7% occurred within 24 hours of surgery, and 77.7% occurred by postoperative day 7. Our findings are helpful for researchers for the planning and conduct of future studies and for clinicians in prevention of surgical complications and in decision-making regarding the start of postsurgical pharmacologic thromboprophylaxis.

## Notes

Supplement 1. **eFigure 1.** Absolute Risk of BIMS, Postoperative Red Blood Cell Transfusion, Postoperative Hemoglobin < 70 and Bleeding Associated With Reintervention on Each Postoperative Day up to Day 30 **eFigure 2.** Absolute Risk of Major Bleeding on Each Postoperative Day up to Day 30 **eFigure 3.** Sensitivity Analysis of Cumulative Proportion of Risk for Major Bleeding by Patient Recruitment Year **eFigure 4.** Sensitivity Analysis of Cumulative Proportion of Risk for Major Bleeding by Surgical Specialty **eTable 1.** Cumulative Proportion of Risk (%) of Events up to Postoperative Day 30 **eTable 2.** Confidence Intervals for Outcomes **eTable 3.** Results of the Secondary Analysis for Late vs Early Postoperative Major Bleeding

Supplement 2.
VISION Investigators Group Members

Supplement 3.
Data Sharing Statement
