# Supplementary material for: Timing of Major Postoperative Bleeding Among Patients Undergoing Surgery
Source: JAMA Netw Open. 2024 Apr 2;7(4):e244581. doi: 10.1001/jamanetworkopen.2024.4581 (PMC10988355; doi:10.1001/jamanetworkopen.2024.4581)
Supplement: Supplement 1. — eFigure 1. Absolute Risk of BIMS, Postoperative Red Blood Cell Transfusion, Postoperative Hemoglobin < 70 and Bleeding Associated With Reintervention on Each Postoperative Day up to Day 30 eFigure 2. Absolute Risk of Major Bleeding on Each Postoperative Day up to Day 30 eFigure 3. Sensitivity Analysis of Cumulative Proportion of Risk for Major Bleeding by Patient Recruitment Year eFigure 4. Sensitivity Analysis of Cumulative Proportion of Risk for Major Bleeding by Surgical Specialty eTable 1. Cumulative Proportion of Risk (%) of Events up to Postoperative Day 30 eTable 2. Confidence Intervals for Outcomes eTable 3. Results of the Secondary Analysis for Late vs Early Postoperative Major Bleeding [file jamanetwopen-e244581-s001.pdf]

Supplemental Online Content

Halme ALE, Roshanov PS, Tornberg SV, Lavikainen LI, Devereaux PJ, Tikkinen KAO; VISION Investigators. Timing of major postoperative bleeding among patients undergoing surgery. *JAMA Netw Open*. 2024;7(4):e244581. doi:10.1001/jamanetworkopen.2024.4581

eFigure 1. Absolute Risk of BIMS, Postoperative Red Blood Cell Transfusion, Postoperative Hemoglobin < 70 and Bleeding Associated With Reintervention on Each Postoperative Day up to Day 30
eFigure 2. Absolute Risk of Major Bleeding on Each Postoperative Day up to Day 30
eFigure 3. Sensitivity Analysis of Cumulative Proportion of Risk for Major Bleeding by Patient Recruitment Year
eFigure 4. Sensitivity Analysis of Cumulative Proportion of Risk for Major Bleeding by Surgical Specialty
eTable 1. Cumulative Proportion of Risk (%) of Events up to Postoperative Day 30
eTable 2. Confidence Intervals for Outcomes
eTable 3. Results of the Secondary Analysis for Late vs Early Postoperative Major Bleeding

This supplemental material has been provided by the authors to give readers additional information about their work.

© 2024 Halme ALE et al. *JAMA Network Open.*

eFigure 1. Absolute Risk of BIMS, Postoperative Red Blood Cell Transfusion, Postoperative Hemoglobin < 70 and Bleeding Associated With Reintervention on Each Postoperative Day up to Day 30

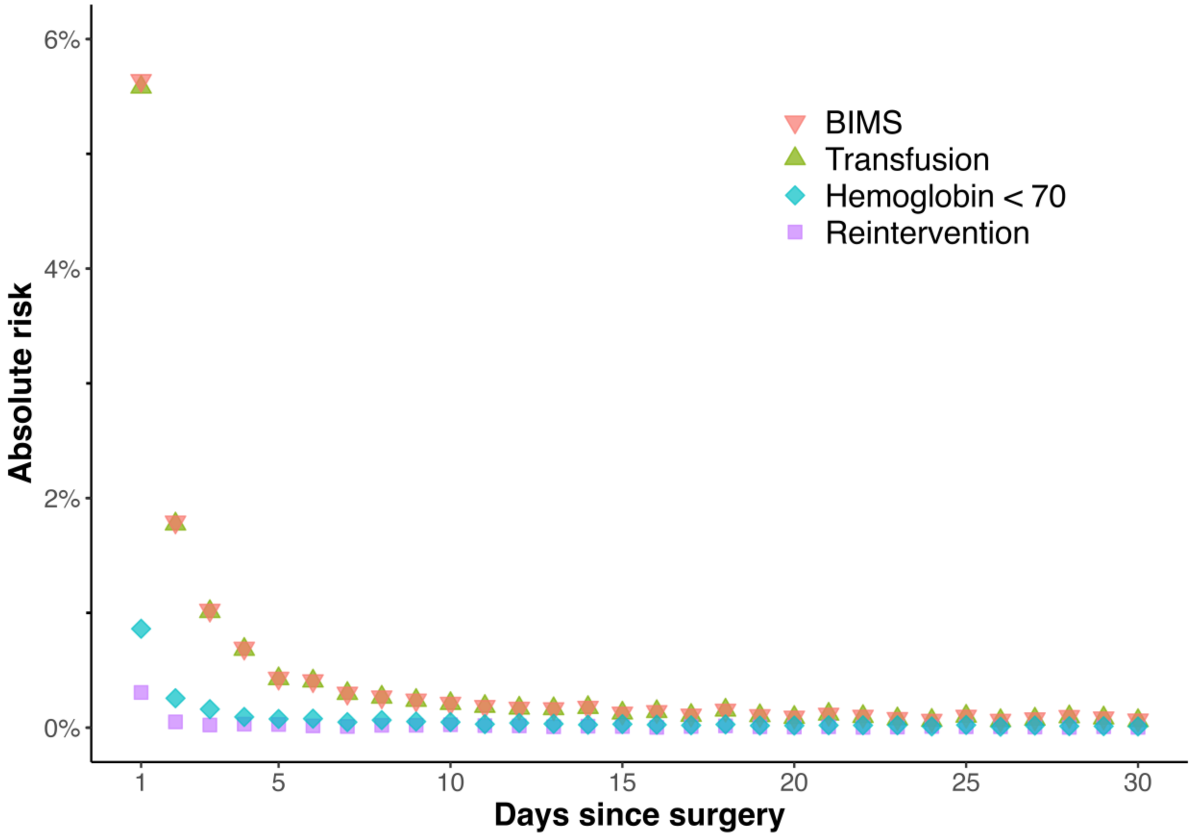

BIMS (Bleeding Independently associated with Mortality after noncardiac Surgery) is defined as a composite of postoperative transfusion, postoperative hemoglobin < 70 and bleeding associated with reintervention.

© 2024 Halme ALE et al. *JAMA Network Open.*

eFigure 2. Absolute Risk of Major Bleeding on Each Postoperative Day up to Day 30

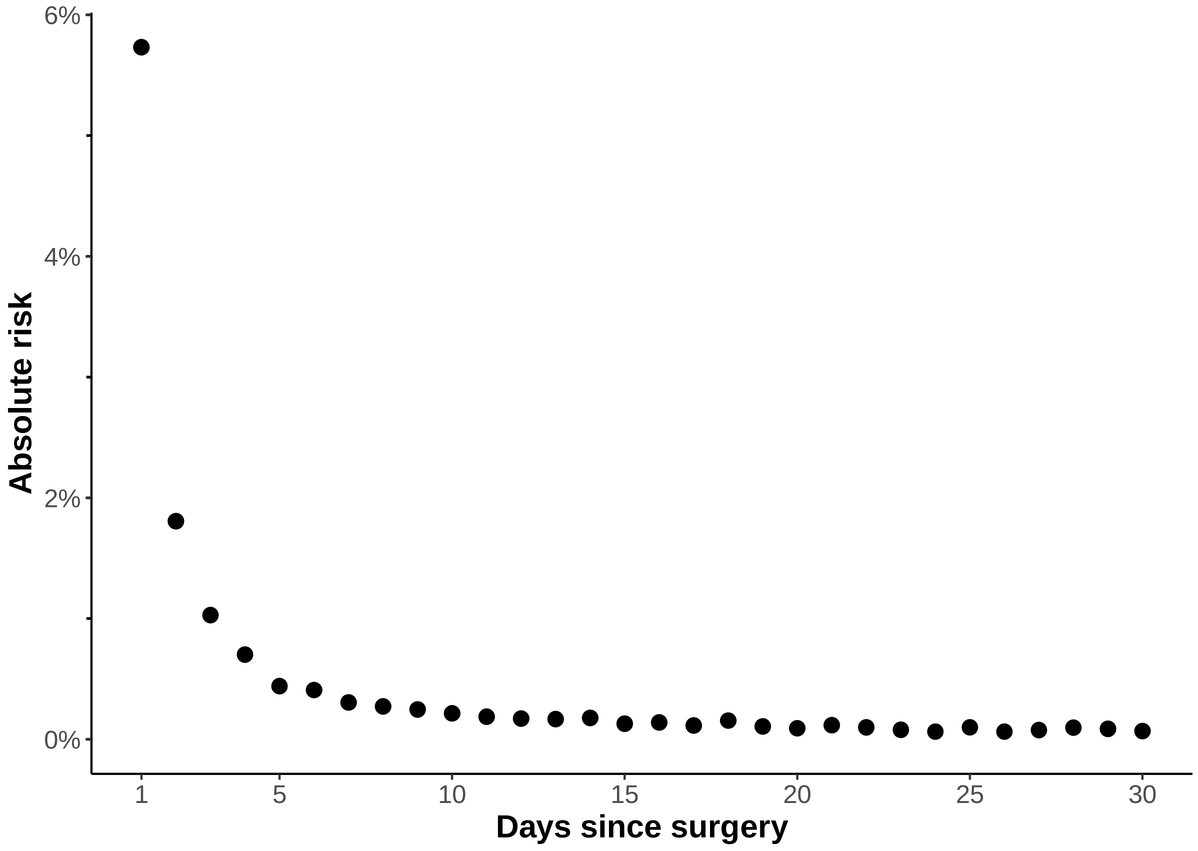

© 2024 Halme ALE et al. *JAMA Network Open.*

eFigure 3. Sensitivity Analysis of Cumulative Proportion of Risk for Major Bleeding by Patient Recruitment Year

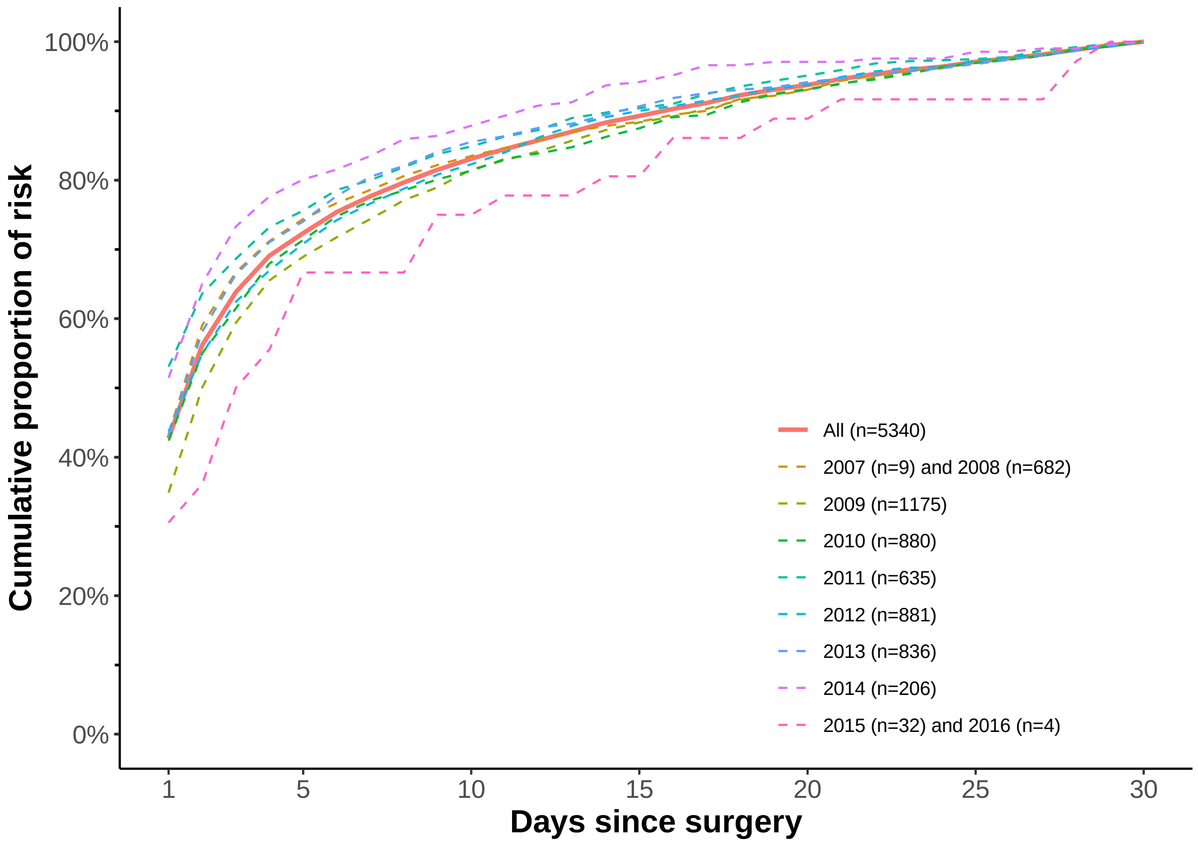

© 2024 Halme ALE et al. *JAMA Network Open.*

eFigure 4. Sensitivity Analysis of Cumulative Proportion of Risk for Major Bleeding by Surgical Specialty

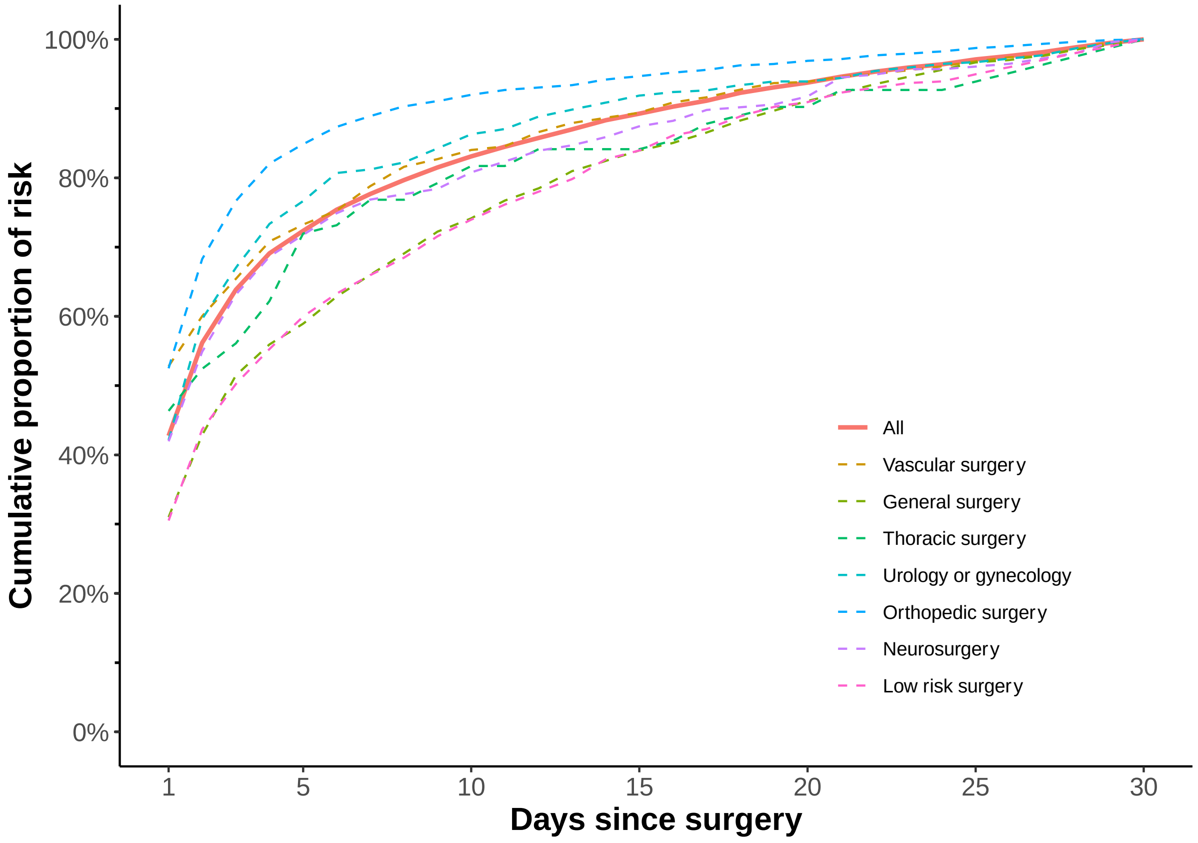

© 2024 Halme ALE et al. *JAMA Network Open.*

eTable 1. Cumulative Proportion of Risk (%) of Events up to Postoperative Day 30

	1 d	2 d	3 d	4 d	5 d	6 d	7 d	14 d	21 d	30 d
Major bleeding*	42.7	56.2	63.9	69.1	72.4	75.4	77.7	88.3	94.6	100
BIMS	42.6	56.1	63.9	69.1	72.3	75.4	77.7	88.3	94.6	100
Transfusion	42.6	56.2	63.9	69.1	72.3	75.4	77.7	88.3	94.6	100
Hg < 70	39.9	51.8	59.3	63.6	67.1	70.7	72.9	86.5	93.8	100
Reintervention	44.5	51.8	55.1	59.5	63.5	65.7	67.2	84.3	92.7	100
Death**	32.0	36.0	40.0	44.0	48.0	52.0	56.0	88.0	92.0	100

*A composite of BIMS and bleeding associated with reintervention
**Very low number of events (n=25)

© 2024 Halme ALE et al. *JAMA Network Open.*

eTable 2. Confidence Intervals for Outcomes

Post-operative day	Lower limit (95% CI)	Risk* (%)	Upper limit (95% CI)
**Major bleeding**			
1	40.9	42.7	44.6
2	54.3	56.2	58.1
3	62.0	63.9	65.7
4	67.2	69.1	70.9
5	70.5	72.4	74.2
6	73.5	75.4	77.3
7	75.8	77.7	79.5
14	86.5	88.3	90.2
21	92.7	94.6	96.5
30		100	
**BIMS**			
1	40.7	42.6	44.5
2	53.4	56.1	58.0
3	62.0	63.9	65.7
4	67.2	69.1	71.0
5	70.5	72.3	74.2
6	73.5	75.4	77.3
7	75.8	77.7	79.5
14	86.4	88.3	90.2
21	92.7	94.6	96.4
30		100	
**Transfusion**			
1	40.7	42.6	44.5
2	54.3	56.2	58.0
3	62.0	63.9	65.8
4	67.2	69.1	71.0
5	70.4	72.3	74.2
6	73.5	75.4	77.3
7	75.8	77.7	79.6
14	86.5	88.3	90.2
21	92.8	94.6	96.5
30		100	
**Hg < 70**			
1	35.3	39.9	44.6
2	47.2	51.8	56.4
3	54.6	59.3	63.9
4	58.9	63.6	68.2
5	62.4	67.1	71.7
6	66.0	70.7	75.3
7	68.2	72.9	77.5
14	81.9	86.5	91.1
21	89.2	93.8	98.5
30		100	
**Reintervention**			
1	36.3	44.5	52.7
2	43.6	51.8	60.0
3	46.9	55.1	63.3
4	51.3	59.5	67.7
5	55.3	63.5	71.7
6	57.5	65.7	73.9
7	58.9	67.2	75.4
14	76.1	84.3	92.5
21	84.5	92.7	100
30		100	

© 2024 Halme ALE et al. *JAMA Network Open.*

Death			
1	4.8	32	59.2
2	8.8	36	63.2
3	12.8	40	67.2
4	16.8	44	71.2
5	20.8	48	75.2
6	24.8	52	79.2
7	28.8	56	83.2
14	60.8	88	100
21	72.8	92	100
30		100	

*Cumulative proportion of risk of the event

© 2024 Halme ALE et al. *JAMA Network Open.*

eTable 3. Results of the Secondary Analysis for Late vs Early Postoperative Major Bleeding

Early bleeding is defined as BIMS or reintervention within the first 2 postoperative days (1) and late as BIMS or reintervention after the first 2 postoperative days (0).

**Characteristic**	**OR**[1]	**95% CI**[1]	**p-value**
Female	1.49	1.28, 1.74	<0.001
**Age**			
45 - 64	—	—	
65 - 79	0.90	0.77, 1.07	0.2
80+	0.87	0.69, 1.09	0.2
**BMI**			
18.5 - 24.9	—	—	
< 18.5	0.76	0.57, 1.02	0.067
25 - 29.9	0.95	0.80, 1.13	0.6
30+	0.81	0.66, 0.99	0.040
**Preoperative hemoglobin**			
>= 130	—	—	
120-130	1.13	0.91, 1.41	0.3
110-120	1.13	0.91, 1.41	0.3
100-110	1.13	0.89, 1.43	0.3

© 2024 Halme ALE et al. *JAMA Network Open.*

Characteristic	OR[1]	95% CI[1]	p-value
90-100	1.07	0.83, 1.38	0.6
80-90	1.41	1.00, 1.99	0.048
70-80	1.39	0.75, 2.58	0.3
<70	1.16	0.45, 3.06	0.8
Tobacco use within 6 months	1.02	0.82, 1.26	0.9
**GFR**			
>=90	—	—	
60-89	0.89	0.72, 1.11	0.3
30-59	0.88	0.67, 1.17	0.4
15-29	0.80	0.49, 1.31	0.4
<15 or dialysis	0.68	0.41, 1.12	0.13
NSAID/COX-2-inhibitor <24h	1.00	0.75, 1.34	>0.9
NSAID/COX-2-inhibitor 7d to >24h	0.97	0.75, 1.26	0.8
ASA, Ticlopidine or Plavix 1d	1.20	0.84, 1.72	0.3
ASA, Ticlopidine or Plavix 7d to >24h	0.90	0.71, 1.15	0.4
Statin <24h	0.88	0.64, 1.20	0.4
Statin 7d to >24h	1.06	0.80, 1.43	0.7

© 2024 Halme ALE et al. *JAMA Network Open.*

Characteristic	OR[1]	95% CI[1]	p-value
Prophylactic SC AA <24h	1.04	0.81, 1.32	0.8
Prophylactic SC AA 7d to >24h	0.66	0.49, 0.90	0.007
DOAC or Therapeutic subc. or IV antithrombotic agent <24h	0.43	0.21, 0.89	0.021
DOAC or Therapeutic subc. or IV antithrombotic agent 7d to >24h	1.31	0.87, 2.00	0.2
**Surgical procedure**			
Major hip or pelvic surgery	—	—	
Low risk surgeries	0.49	0.38, 0.64	<0.001
Thoracic aorta reconstruction	0.69	0.24, 2.03	0.5
Aorta-iliac reconstruction	1.12	0.66, 1.95	0.7
Peripheral vascular reconstruction without aortic cross-clamping	0.68	0.43, 1.10	0.12
Extracranial cerebrovascular surgery	0.51	0.19, 1.40	0.2
EVAR	0.89	0.40, 2.06	0.8
Complex visceral resection	0.46	0.32, 0.65	<0.001
Partial or total colectomy, or stomach surgery	0.48	0.35, 0.64	<0.001
Other intra-abdominal surgery	0.37	0.26, 0.52	<0.001

© 2024 Halme ALE et al. *JAMA Network Open.*

Characteristic	OR[1]	95% CI[1]	p-value
Major head and neck resection for non-thyroid tumor	1.18	0.68, 2.08	0.6
Lobectomy	0.33	0.13, 0.82	0.016
Other thoracic surgery	0.76	0.35, 1.78	0.5
Visceral resection	0.80	0.50, 1.28	0.3
Cytoreductive surgery	0.22	0.11, 0.45	<0.001
Hysterectomy	1.36	0.71, 2.81	0.4
Radical hysterectomy	0.86	0.31, 2.62	0.8
Radical prostatectomy	1.09	0.50, 2.53	0.8
Transuretheral prostatectomy	0.99	0.49, 2.05	>0.9
Internal fixation of femur	1.10	0.75, 1.62	0.6
Knee arthroplasty	1.31	0.98, 1.74	0.067
Above knee amputation	0.64	0.35, 1.18	0.15
Lower leg amputation	0.68	0.36, 1.30	0.2
Craniotomy	0.62	0.33, 1.15	0.12
Major spine surgery	1.02	0.65, 1.59	>0.9
**Recruitment year**			
2009	—	—	

© 2024 Halme ALE et al. *JAMA Network Open.*

Characteristic	OR[1]	95% CI[1]	p-value
2007	0.14	0.01, 0.92	0.079
2008	1.03	0.79, 1.36	0.8
2010	1.37	1.05, 1.79	0.021
2011	1.30	0.94, 1.81	0.12
2012	1.02	0.75, 1.38	>0.9
2013	1.18	0.86, 1.62	0.3
2014	1.28	0.79, 2.09	0.3
2015	0.27	0.10, 0.71	0.008
2016	1.53	0.06, 40.7	0.8
**Study center**			
2	—	—	
1	0.69	0.36, 1.37	0.3
3	0.25	0.15, 0.43	<0.001
4	1.67	0.90, 3.29	0.12
5	0.27	0.18, 0.42	<0.001
6	1.49	0.88, 2.60	0.15
10	0.39	0.28, 0.54	<0.001

© 2024 Halme ALE et al. *JAMA Network Open.*

Characteristic	OR[1]	95% CI[1]	p-value
11	0.67	0.37, 1.25	0.2
21	0.19	0.12, 0.32	<0.001
22	0.21	0.10, 0.44	<0.001
31	0.84	0.44, 1.65	0.6
35	0.18	0.07, 0.45	<0.001
40	0.31	0.21, 0.45	<0.001
41	0.35	0.24, 0.51	<0.001
42	0.22	0.14, 0.33	<0.001
43	0.14	0.07, 0.27	<0.001
45	0.52	0.21, 1.37	0.2
46	0.50	0.29, 0.90	0.020
49	0.20	0.11, 0.38	<0.001
51	0.29	0.19, 0.44	<0.001
52	0.38	0.25, 0.58	<0.001
60	0.29	0.19, 0.47	<0.001
80	0.23	0.14, 0.38	<0.001
81	1.73	0.96, 3.30	0.079

© 2024 Halme ALE et al. *JAMA Network Open.*

Characteristic	OR[1]	95% CI[1]	p-value
82	2.69	1.42, 5.31	0.003
90	0.54	0.19, 1.64	0.3
92	0.61	0.37, 1.02	0.055

OR, odds ratio; CI, Confidence Interval; GFR, glomerular filtration rate; NSAID, nonsteroidal anti-inflammatory drug; COX-2, cyclooxygenase-2; BMI, body mass index; ASA, acetylsalicylic acid, SC AA, subcutaneous antithrombotic agent, DOAC, direct oral anticoagulant; EVAR, endovascular aneurysm repair

Study center 36 (n = 1) and pneumonectomy (n = 3) were excluded from the table due to low event counts. The OR, 95% CI and p-value for them is available from the corresponding author upon reasonable request.

BIMS (Bleeding Independently associated with Mortality after noncardiac Surgery) is defined as a composite of postoperative transfusion, postoperative hemoglobin < 70 and bleeding associated with reintervention.

© 2024 Halme ALE et al. *JAMA Network Open.*
