# Supplementary material for: Timing of Major Postoperative Bleeding Among Patients Undergoing Surgery
Source: JAMA Netw Open. 2024 Apr 2;7(4):e244581. doi: 10.1001/jamanetworkopen.2024.4581 (PMC10988355; doi:10.1001/jamanetworkopen.2024.4581)
Supplement: Supplement 2. — VISION Investigators Group Members [file jamanetwopen-e244581-s002.pdf]

Supplemental Online Content: Nonauthor Collaborators
*First name, last name, and suffix (if applicable) are required and will appear in PubMed.

*Group Name(s): VISION investigators							
***First Name and Middle Initial(s)**	***Last Name**	***Suffix (eg, Jr, III)**	Academic Degrees	Institution	Location (city, state/province, country)	Role or Contribution, eg, chair, principal investigator	Group (if more than 1 Group listed in the byline) and/or Subgroup (eg, Steering Committee)
Justin	DeBeer						
Clive	Kearon						
Richard	Mizera						
Jehonathan	Pinthus						
Sebastian	Ribas						
Tej	Sheth						
Marko	Simunovic						
Vikas	Tandon						
Tomas	VanHelder						
Mitchell	Winemaker						
James	Paul						
Zubin	Punthakee						
Karen	Raymer						
Anthony	Adili						
Catherine	Clase						
Deborah	Cook						
James	Douketis						
Azim	Gangji						
Paul	Jackson						
Wendy	Lim						
Peter	Lovrics						
Sergio	Mazzadi						
Maria	Tiboni						
John	Eikelboom						
Javier	Ganame						
James	Hankinson						
Stephen	Hill						
Sanjit	Jolly						

Supplemental Online Content: Nonauthor Collaborators
*First name, last name, and suffix (if applicable) are required and will appear in PubMed.

***First Name and Middle Initial(s)**	***Last Name**	***Suffix (eg, Jr, III)**	Academic Degrees	Institution	Location (city, state/province, country)	Role or Contribution, eg, chair, principal investigator	Group (if more than 1 Group listed in the byline) and/or Subgroup (eg, Steering Committee)
Elizabeth	Ling						
Patrick	Magloire						
Guillaume	Pare						
David	Szalay						
Jacques	Tittley						
Omid	Salehian						
Hertzel	Gerstein						
Sadeesh K	Srinathan						
Clare	Ramsey						
Philip	St. John						
Laurel	Thorlacius						
Faisal S	Siddiqui						
Hilary P	Grocott						
Andrew	McKay						
Trevor WR	Lee						
Ryan	Amadeo						
Duane	Funk						
Heather	McDonald						
James	Zacharias						
Rey	Acedillo						
Amit	Garg						
Ainslie	Hildebrand						
Ngan	Lam						
Danielle	MacNeil						
Marko	Mrkobrada						
Pavel	Roshanov						
Daniel I	Sessler						
Andrea	Kurz						
Emre	Gorgun						
Amanda	Naylor						

Supplemental Online Content: Nonauthor Collaborators
*First name, last name, and suffix (if applicable) are required and will appear in PubMed.

*First Name and Middle Initial(s)	*Last Name	*Suffix (eg, Jr, III)	Academic Degrees	Institution	Location (city, state/province, country)	Role or Contribution, eg, chair, principal investigator	Group (if more than 1 Group listed in the byline) and/or Subgroup (eg, Steering Committee)
Matt	Hutcherson						
Zhuo	Sun						
Bianka	Nguyen						
Michael	Palma						
Avis	Cuko						
Aram	Shahinyan						
Vinayak	Nadar						
Mauricio	Perilla						
Kamal	Maheshwari						
Alparslan	Turan						
Rupert	Pearse						
Edyta	Niebrzegowska						
Andrew	Wrag						
Andrew	Archbold						
Elisa	Kam						
Kirsty	Everingham						
Phoebe	Bodger						
Thais	Creary						
Ben	Bloom						
Alice	Carter						
Tom E F	Abbott						
Nirav	Shah						
Katarzyna	Mrozek						
Amy	Richardson						
Alex	Fowler						
Zakaria	Rob						
Gareth	Ackland						
Robert	Stephens						
Anna	Reyes						
Laura	Gallego Paredes						

Supplemental Online Content: Nonauthor Collaborators
*First name, last name, and suffix (if applicable) are required and will appear in PubMed.

***First Name and Middle Initial(s)**	***Last Name**	***Suffix (eg, Jr, III)**	Academic Degrees	Institution	Location (city, state/province, country)	Role or Contribution, eg, chair, principal investigator	Group (if more than 1 Group listed in the byline) and/or Subgroup (eg, Steering Committee)
Pervez	Sultan						
David	Cain						
John	Whittle						
Ana	Gutierrez del Arroyo						
Shamir	Karmali						
C	Williams						
A	Rushton						
I	Welters						
M	Leuwer						
Jane	Parker						
Robert J	Sapsford						
Julian	Barth						
Julian	Scott						
Alistair	Hall						
Simon	Howell						
Michaela	Lobley						
Janet	Woods						
Susannah	Howard						
Joanne	Fletcher						
Nikki	Dewhirst						
Wojciech	Szczeklik						
Jacek	Gorka						
Karolina	Gorka						
Bogusz	Kaczmarek						
Kamil	Polok						
Jolanta	Gasior						
Anna	Włudarczyk						
Magdalena	Duchińska						
Jakub	Fronczek						
Aleksandra	Wojnarska						

Supplemental Online Content: Nonauthor Collaborators
*First name, last name, and suffix (if applicable) are required and will appear in PubMed.

***First Name and Middle Initial(s)**	***Last Name**	***Suffix (eg, Jr, III)**	Academic Degrees	Institution	Location (city, state/province, country)	Role or Contribution, eg, chair, principal investigator	Group (if more than 1 Group listed in the byline) and/or Subgroup (eg, Steering Committee)
Mateusz	Kozka						
Andrzej	Halek						
Pierre	Coriat						
Denis	Monneret						
Marie-Hélène	Fléron						
Jean Pierre	Goarin						
Cristina	Ibanez Esteve						
Catherine	Royer						
Georges	Daas						
Valsa	Abraham						
Preetha	George						
Denis	Xavier						
Alben	Sigamani						
Atiya	Faruqui						
Radhika	Dhanpal						
Smitha	Almeida						
Joseph	Cherian						
Sultana	Furruqh						
CY	Wang						
GSY	Ong						
M	Mansor						
Alvin SB	Tan						
II	Shariffuddin						
NHM	Hashim						
A Wahab	Undok						
HY	Lai						
WAW	Ahmad						
PS	Loh						
CY	Chong						
AHA	Razack						

Supplemental Online Content: Nonauthor Collaborators
*First name, last name, and suffix (if applicable) are required and will appear in PubMed.

*First Name and Middle Initial(s)	*Last Name	*Suffix (eg, Jr, III)	Academic Degrees	Institution	Location (city, state/province, country)	Role or Contribution, eg, chair, principal investigator	Group (if more than 1 Group listed in the byline) and/or Subgroup (eg, Steering Committee)
Matthew TV	Chan						
Gordon YS	Choi						
Lydia CW	Lit						
Tony	Gin						
Alex	Wan						
Linda	Lai						
Polly	Chan						
German	Malaga						
Vanessa	Valderrama-Victoria						
Javier D	Loza-Herrera						
Maria	De Los Angeles Lazo						
Aida	Rotta-Rotta						
Otavio	Berwanger						
Erica	Suzumura						
Eliana	Santucci						
Katia	Leite						
Jose	Amalth do Espirirto Santo						
Cesar AP	Jardim						
Alexandre	Biasi Cavalcanti						
Helio Penna	Guimaraes						
Carisi A	Polanczyk						
Mariana V	Furtado						
Olga Lucía	Cortés						
Félix R	Montes						
Paula A	Alvarado						
Juan Carlos	Villar						
Skarlett	Vásquez						
Bruce	Biccard						
Hussein	Cassimjee						

Supplemental Online Content: Nonauthor Collaborators
*First name, last name, and suffix (if applicable) are required and will appear in PubMed.

***First Name and Middle Initial(s)**	***Last Name**	***Suffix (eg, Jr, III)**	Academic Degrees	Institution	Location (city, state/province, country)	Role or Contribution, eg, chair, principal investigator	Group (if more than 1 Group listed in the byline) and/or Subgroup (eg, Steering Committee)
Dean	Gopalan						
Theroshnie	Kisten						
Aine	Mugabi						
Prebashini	Naidoo						
Rubeshan	Naidoo						
Reitze	Rodseth						
David	Skinner						
Alex	Torborg						
Clara K	Chow						
Graham S	Hillis						
Richard	Halliwell						
Stephen	Li						
Vincent W	Lee						
John	Mooney						
