# Supplementary material for: Timing of Major Postoperative Bleeding Among Patients Undergoing Surgery
Source: JAMA Netw Open. 2024 Apr 2;7(4):e244581. doi: 10.1001/jamanetworkopen.2024.4581 (PMC10988355; doi:10.1001/jamanetworkopen.2024.4581)
Supplement: Supplement 3. — Data Sharing Statement [file jamanetwopen-e244581-s003.pdf]

Data Sharing Statement

Halme. Timing of Major Postoperative Bleeding Among Patients Undergoing Surgery. *JAMA Netw Open*. Published April 02, 2024. doi:10.1001/jamanetworkopen.2024.4581

Data
Data available: No

Additional Information
Explanation for why data not available: The datasets generated during and/or analyzed during the current study are not publicly available but will be made available to journal statisticians as required.
